# ABRLog: a multi-device android bugreport log dataset for intelligent analysis

**DOI:** 10.1016/j.dib.2026.113230

**Published:** 2026-09-07

**Authors:** Ricardo Miranda Filho, João Alfredo Bessa, Rosiane de Freitas

**Affiliations:** PPGI - Instituto de Computação, Universidade Federal do Amazonas (IComp/UFAM), Av. General Rodrigo Octávio 6200, Coroado I, 69080-900, Manaus - AM, Brazil

**Keywords:** Android logs, Anomaly detection, Bug report, Data science, Log analysis, Machine learning, Mobile computing

## Abstract

Android devices generate a wealth of diagnostic data through their built-in logging subsystems, offering invaluable insights into application behavior, hardware performance, and operating system stability. However, the effective utilization of this data for intelligent analysis — particularly, for anomaly detection, failure diagnosis, and security monitoring — remains constrained by the scarcity of publicly available, well-structured, and multi-device log datasets. To address this gap, we introduce ABRLog (Android BugReport Log), a comprehensive multi-device log dataset derived from bug report captures collected across 15 distinct Android devices representing five major manufacturers (Samsung, Motorola, Google, Xiaomi, and Huawei) and spanning six Android versions, from 9 to 14. A total of 45 bugreport archives were systematically processed through two complementary extraction strategies: (i) line-by-line regular expression matching for granular event extraction; and (ii) section-based delimiter parsing for high-level block segmentation. This dual approach yielded 1012 structured log files, organized into 24 semantically coherent block types. The final dataset comprises approximately 10 million log lines (1.14 GiB), extracted from 6.34 GiB of raw bugreport data. By providing a systematically curated, multi-vendor, multi-version log corpus, ABRLog aims to serve as a foundational resource for the development and benchmarking of machine learning models for log-based anomaly detection, predictive maintenance, and intelligent system diagnostics on Android platforms.

Specifications Table

**Table:** 

Subject	Computer Science, Machine Learning
Specific subject area	Intelligent log analysis, mobile computing, anomaly detection, system diagnostics
Type of data	Text files (structured log blocks), CSV (parsed logs and templates), tables, figures, Python scripts
Data collection	Data were collected manually from 15 Android smartphones using the BugReport tool via developer options. Two extraction methods were applied: (1) line-by-line regular expression matching for structured log formats, and (2) section-based delimiter parsing for bugreport sections demarcated by standard headers. The Drain log parser was used to generate templates and structured CSV files. Personal identifiers present in the captures were pseudonymized before publication; see the Ethics Statement.
Data source location	Institution: Universidade Federal do Amazonas City/State: Manaus - Amazonas Country: Brazil
Data accessibility	Repository name: Zenodo – ABRLog Dataset Data identification number: 10.5281/zenodo.21892365 Direct URL: https://doi.org/10.5281/zenodo.21892365
Related research article	J. A. Bessa, G. Souza, L. Pessoa, R. Barreto, R. de Freitas et al., “Análise de desempenho de log parsers da coleção LogPAI em dados brutos de dispositivos Android”. Workshop em Desempenho de Sistemas Computacionais e de Comunicação (WPerformance), SBC, 2024, pp. 37–48. J. A. Bessa, R. Miranda Filho, G. Souza, R. Barreto, and R. de Freitas, “Log parsers performance on raw logs from Android devices”. Journal of Internet Services and Applications (JISA), vol. 16, no. 1, pp. 105–116, 2025. DOI: https://doi.org/10.5753/jisa.2025.5049 J. A. Bessa, R. Miranda Filho, R. Barreto, and R. de Freitas, “A multi-device Android mobile raw log dataset for intelligent analysis”. Brazilian Symposium on Computing Systems Engineering (SBESC), 2025, pp. 1–6.

## 1 Value of the Data

These data are primarily intended for three research communities: (i) researchers who design and benchmark log parsers, who need heterogeneous real-world input beyond the server-oriented corpora currently available; (ii) mobile systems and digital forensics researchers, who require full-device diagnostic snapshots rather than a single log stream; and (iii) machine learning practitioners working on anomaly detection and failure prediction over operational telemetry.

• **Multi-manufacturer variability.** The dataset covers five Android manufacturers (Samsung, Motorola, Google, Xiaomi, and Huawei) across 15 devices, exposing the lack of standardization in log formatting and organization across the Android ecosystem. This variability is essential for evaluating the robustness of log analysis tools.
• **Comprehensive block segmentation.** Unlike existing log datasets that typically provide a single log stream, ABRLog offers 24 distinct block types extracted from bugreports, including system logs, kernel logs, battery statistics, binder IPC data, network statistics, and process listings. This granularity enables subsystem-level analysis of the Android architecture. Compared with the closest mobile comparator, ChiDroid [1], which collects a single logcat stream from two devices, ABRLog provides 24 block types from 15 devices of five manufacturers (Table 3), supporting reuse scenarios—such as cross-subsystem correlation and cross-manufacturer generalization—that a single-stream dataset cannot address.
• **Scale and temporal coverage.** With 45 bugreport captures totaling approximately 10 million lines of structured logs, the dataset provides sufficient volume for training and evaluating machine learning models. Multiple captures per device enable temporal analysis of device behavior.
• **Parsed log references.** The dataset includes Drain-generated templates and structured parsed logs for seven major block types, providing reference output for benchmarking log parsing algorithms on real-world Android data. These references are produced automatically and were not manually verified; they constitute a reproducible baseline rather than a manually curated ground truth (see Limitations).
• **Reproducible and extensible pipeline.** The open-source Python extraction scripts support both regex-based and section-based extraction methods. New block types can be added by defining new patterns or section keywords, and the parsing pipeline can be extended to additional log parsers.

## 2 Background

Logs in mobile devices systematically capture events, system activities, and application behavior, offering critical insights into performance, diagnostics, and user interaction. These records include system-level events, application-specific messages, and error logs, typically enriched with timestamps, event metadata, and contextual information. Their analysis enables developers, administrators, and researchers to identify performance issues, usage patterns, and potential system failures [2,3].

The proliferation of Android devices has made system logs integral to mobile computing research, serving as a primary data source for fault detection, performance optimization, and security analysis [1,4]. However, effective analysis is hindered by diverse formats and the unstructured nature of these logs across different device manufacturers and Android versions. Each manufacturer implements its own logging conventions, resulting in significant variability in how log data is organized within bugreport captures.

Android’s bugreport tool, accessible through developer options, generates comprehensive diagnostic dumps that include kernel messages, system logs, battery statistics, process information, network state, and dozens of other system-level data sections. These bugreport files typically range from 50 MB to 500 MB per capture, containing hundreds of thousands of lines of mixed log data. While this richness makes bugreports valuable for diagnostics, the lack of standardization across devices creates challenges for automated analysis.

Existing log datasets in the literature, such as the Loghub collection [2], primarily focus on server-side systems (HDFS, Hadoop, BGL) and provide single-type log streams. Datasets specifically targeting Android mobile device logs, particularly those capturing the full diversity of bugreport sections across multiple manufacturers, remain scarce. ABRLog was compiled to address this gap, providing structured log blocks and parsed references extracted from raw bugreport captures across a diverse set of Android devices.

This dataset was originally motivated by the need to evaluate log parser performance on real-world mobile data, as reported in our related studies [5,6]. The current expanded version significantly broadens the scope by increasing the number of devices from 7 to 15, manufacturers from 3 to 5, and extracted block types from 8 to 24, while also adding Drain-based parsed log references. This data article directly extends the collection first described at SBESC 2025 [6], from which it inherits the extraction methodology; the description of the Android logging subsystem summarized above is developed at length in that companion paper and is reproduced here only to the extent needed to make the present data article self-contained.

The ABRLog dataset is one of the outcomes of the SWPERFI (Software Performance Intelligence) Research, Development, and Innovation (RD&I) project, a partnership between the Federal University of Amazonas (UFAM) and Motorola Mobility LLC [7]. This project explores advanced data mining, optimization, and artificial intelligence techniques for software performance analysis. Within the same collaboration, the MUVY (A multi-view user-generated sports video dataset) was also developed, focusing on user-generated sports videos from multiple perspectives, demonstrating the project's broad scope, ranging from system-level log analysis to computer vision applications [8,9].

## 3 Data Description

ABRLog consists of logs extracted from 15 Android devices, organized into raw bugreport captures, structured log blocks, and parsed log references. Table 1 provides an overview of the devices, Table 2 summarizes the extracted block types, and Figs. 1 through 6 present visual analyses of the dataset.

**Table 1:** Overview of Android devices in ABRLog, grouped by manufacturer. Sizes are given in binary units (1 MiB = 1024 KiB). Commercial model numbers and firmware builds of each device are given in Table 6.

Manufacturer	Device	Android	SDK	Bugreports	Raw Size (MiB)
Google	Pixel 6 Pro	13	33	5	241.3
	Pixel 7 Pro	13	33	1	69.0
Huawei	HUAWEI L29	12	31	1	71.3
Motorola	Moto G200	12	31	4	1528.9
	Moto G30	12	31	11	1030.0
	Motorola G53	14	34	1	126.0
	Motorola G71	12	31	8	1487.3
Samsung	Galaxy A8	9	28	3	184.8
	Galaxy S9	9	28	1	78.9
	Galaxy A70	11	30	1	138.4
	Galaxy A22	13	33	1	130.6
	Galaxy S22+	13	33	4	774.2
	Galaxy S21 FE	14	34	2	196.8
	Galaxy S23+	14	34	1	300.2
Xiaomi	POCO X5	14	34	1	131.5
**manufacturers**	**15 devices**	**9–14**	**28–34**	**45**	**6489.2**

**Table 2:** Extracted block types with descriptions and statistics, grouped by extraction method. Coverage indicates the percentage of bugreports from which the block was successfully extracted.

Method	Block Type	Description	Files	Lines	Size (MiB)	Cov. (%)
regex	logcat	System and app log messages	45	4593,130	664.54	100.0
	batterystats	Battery state transitions	45	1472,653	120.07	100.0
	dmesg	Kernel ring buffer messages	44	1137,480	95.54	97.8
	wakelock	Wake lock acquire/release events	41	222,606	13.32	91.1
	system_properties	Android system key-value props	45	70,785	2.91	100.0
	cpuinfo	Per-process CPU usage	45	7636	0.47	100.0
	memory	/proc/meminfo entries	45	1991	0.06	100.0
	battery_capacity	Battery capacity summaries	31	31	0.00	68.9
section	binder_state	Binder IPC transaction state	44	737,436	52.21	97.8
	event_log	Structured event log entries	45	407,697	56.66	100.0
	kernel_log	Kernel log section	45	382,252	33.96	100.0
	radio_log	Radio interface layer log	45	380,865	85.35	100.0
	binder_stats	Binder IPC usage statistics	44	288,889	5.81	97.8
	processes	Running processes and threads	45	208,679	29.58	100.0
	network_stats	Network interface statistics	45	33,216	2.54	100.0
	iptables	Firewall rules dump	45	6485	0.46	100.0
	last_logcat	Last logcat before crash	45	6044	0.74	100.0
	vm_stats	Virtual memory statistics	45	5368	0.12	100.0
	log_statistics	Per-tag log volume statistics	45	3446	0.22	100.0
	interrupts	Hardware interrupt counters	10	2233	0.28	22.2
	filesystems	Mounted filesystems and space	45	1914	0.15	100.0
	psi_io	I/O pressure stall information	41	123	0.00	91.1
	psi_memory	Memory pressure stall info	41	123	0.00	91.1
	psi_cpu	CPU pressure stall information	41	83	0.00	91.1
	**Total**		**1012**	**9971,165**	**1164.99**

**Fig. 1:**
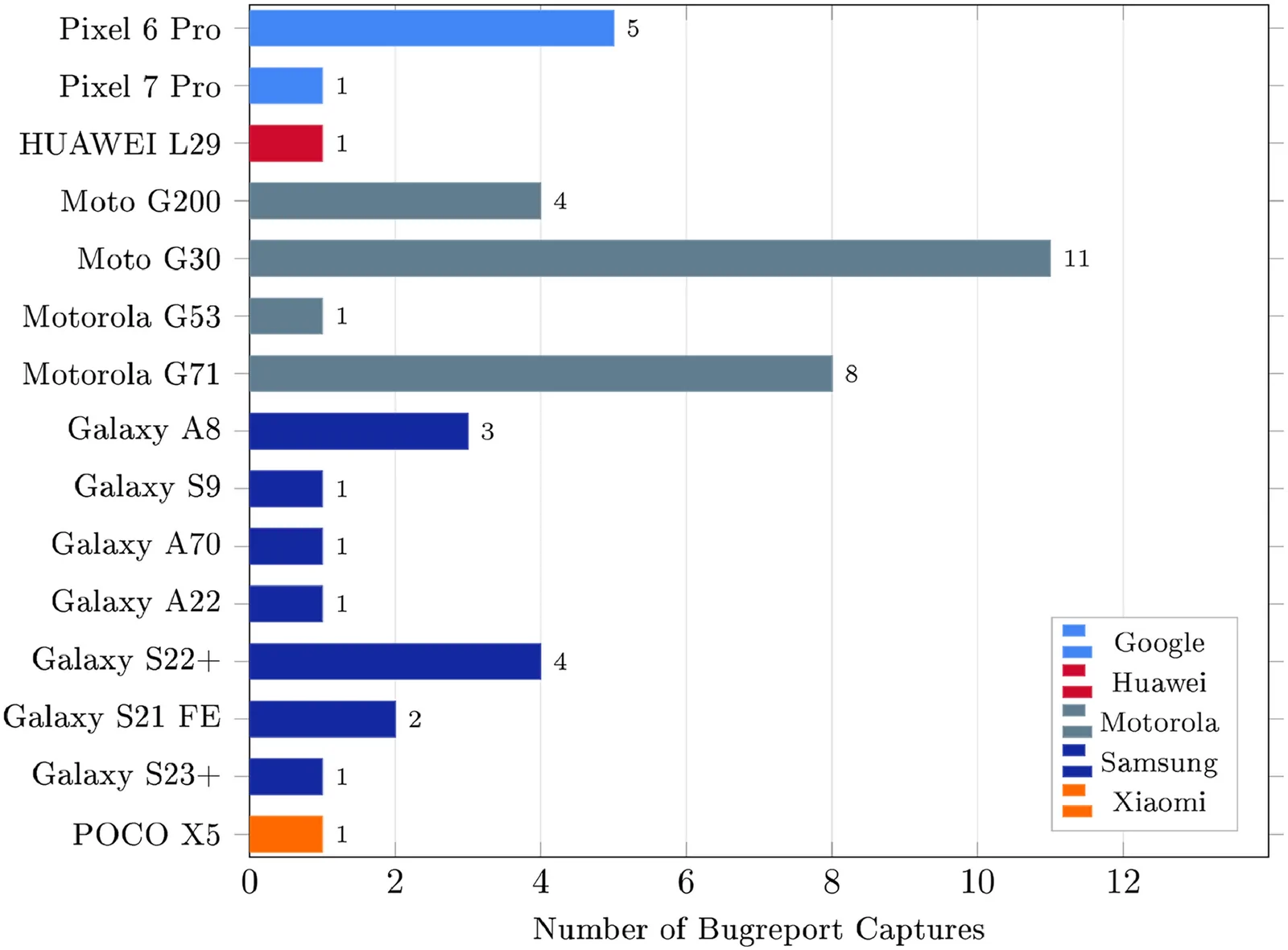
Distribution of bugreport captures per device, color-coded by manufacturer.

### 3.1 Device overview

The dataset covers 15 devices from five manufacturers, spanning Android versions 9–14 (API levels 28–34). Table 1 lists each device, its manufacturer, Android version, number of bugreport captures, and raw data size. The devices range from budget models (Samsung Galaxy A8, Galaxy A22) to flagship devices (Samsung Galaxy S23+, Google Pixel 7 Pro), offering a range of hardware capabilities and software configurations.

Fig. 1 shows the distribution of bugreport captures across devices, color-coded by manufacturer. The Moto G30 contributes the most captures (11), followed by the Motorola G71 (8) and the Google Pixel 6 Pro (5).

### 3.2 Extracted block types

A total of 24 block types were extracted using two complementary methods: line-by-line regular expression matching (8 types) and section-based delimiter parsing (16 types). Table 2 presents all extracted block types grouped by extraction method, along with a brief description, file counts, line totals, sizes, and coverage percentages. Coverage (Cov.) is defined as the number of captures in which the block type is present divided by the 45 captures of the dataset, expressed as a percentage; a block is counted as present when the corresponding file was created and is non-empty. The complete set of extraction rules—the 8 regular expressions and the 17 section delimiters—is listed in Table 7, so that the pipeline can be reproduced from the manuscript alone. The CSV schemas of the parsed references derived from these blocks are defined in the Data Records section.

The most voluminous block types are logcat (4.6 million lines, 46.1% of all extracted data), batterystats (1.5 million lines, 14.8%), and dmesg (1.1 million lines, 11.4%). Fig. 2 shows sample excerpts from six representative block types, illustrating the diversity of log formats present in the dataset.

**Fig. 2:**
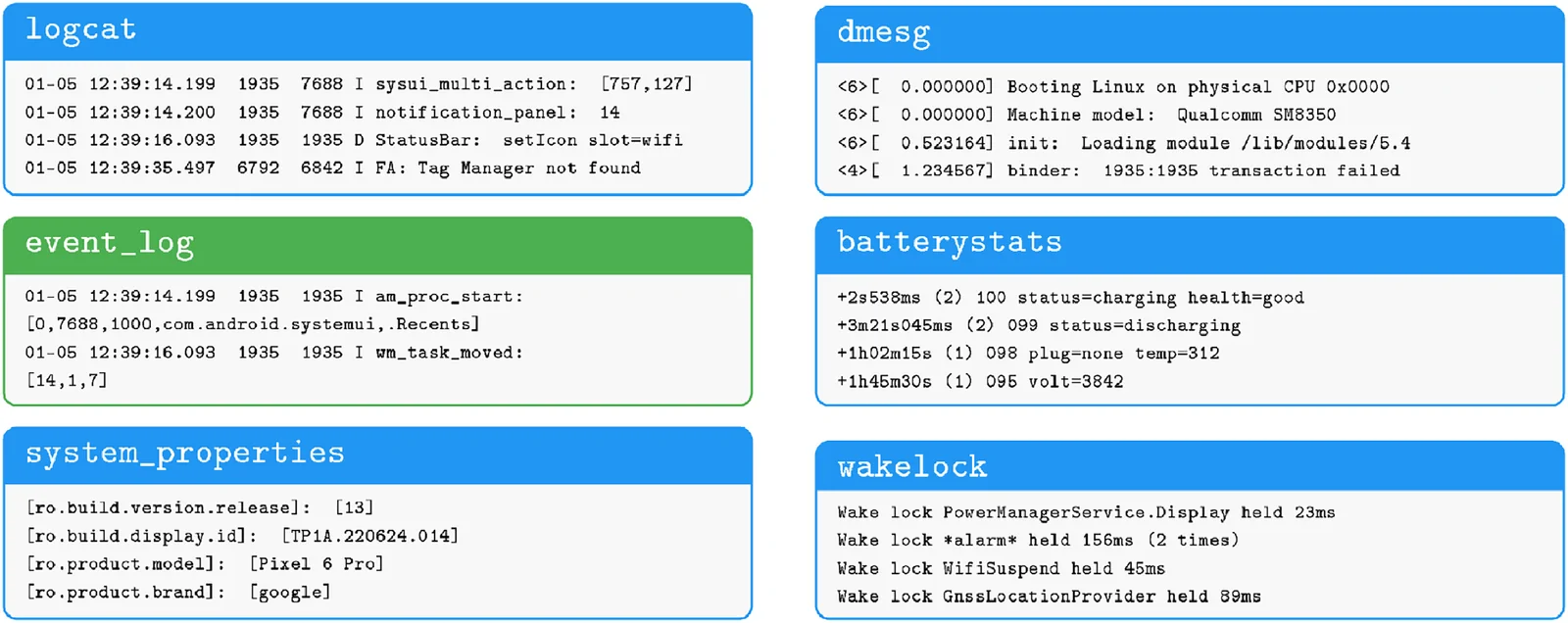
Sample log excerpts from six representative block types in ABRLog. Blue borders indicate regex-extracted blocks; green borders indicate section-based extraction.

As the excerpts in Fig. 2 illustrate, log formats vary considerably across block types—from timestamped free-text messages in logcat to structured key-value pairs in system_properties and tabular process listings in processes. This heterogeneity motivates the use of two distinct extraction strategies and underscores the analytical richness of the dataset. Fig. 3 presents the distribution of extracted lines across all 24 block types on a logarithmic scale, revealing a dynamic range spanning four orders of magnitude—from 31 lines (battery_capacity) to 4.6 million (logcat)—and color-codes each block by its extraction method.

**Fig. 3:**
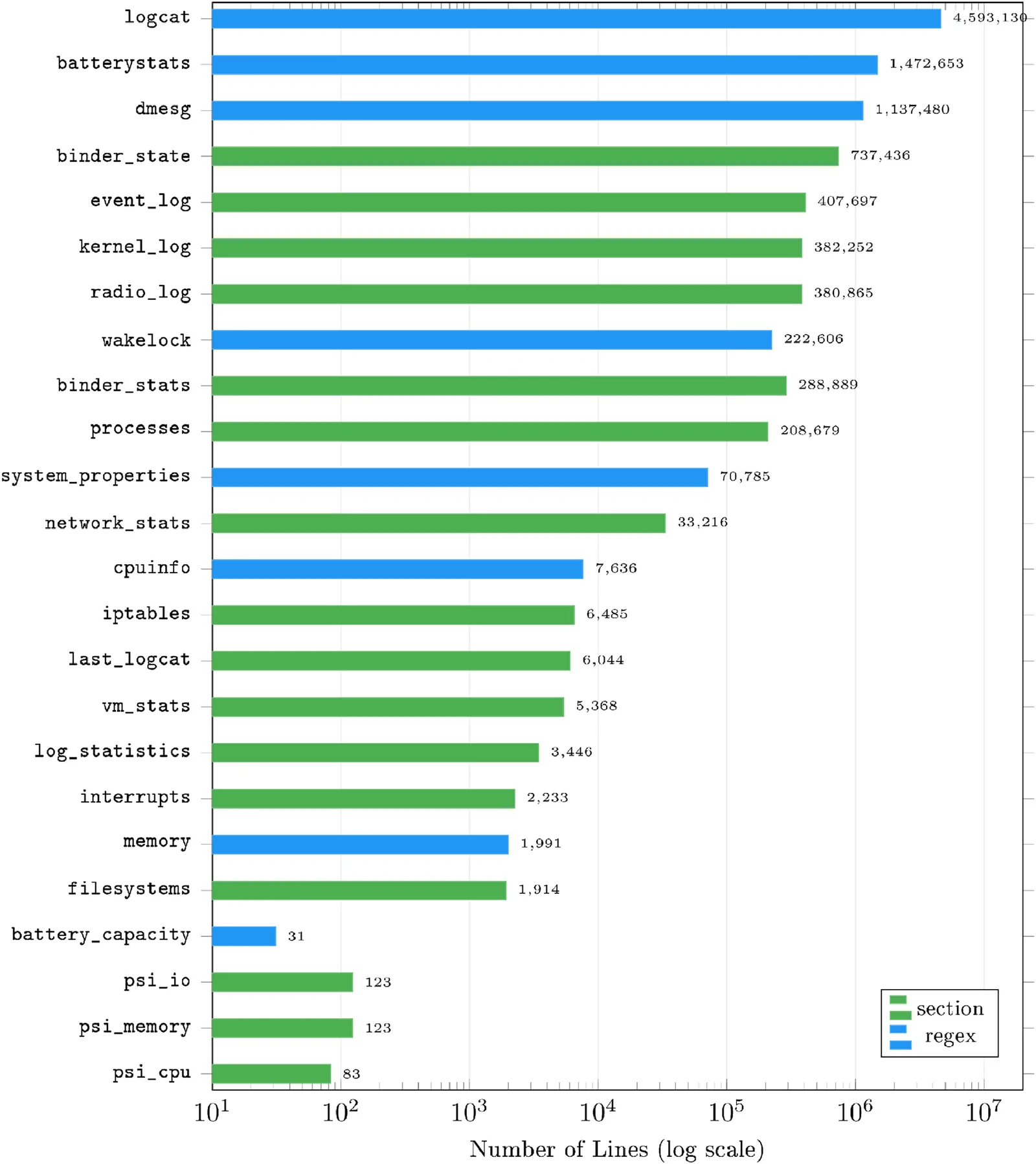
Total lines extracted per block type (logarithmic scale), color-coded by extraction method: regex (blue) and section-based (green).

### 3.3 Coverage analysis

Most block types achieve 100% coverage across all 45 bugreport captures. The interrupts block has the lowest coverage (22.2%), as it is present exclusively in Samsung devices. The battery_capacity block (68.9% coverage) is absent from some Samsung devices that use a different battery reporting format. The Pressure Stall Information (PSI) blocks achieve 91.1% coverage, as they are unavailable on Android versions prior to SDK 30.

Fig. 4 shows the block coverage matrix across all devices, revealing manufacturer-specific differences in bugreport content.

**Fig. 4:**
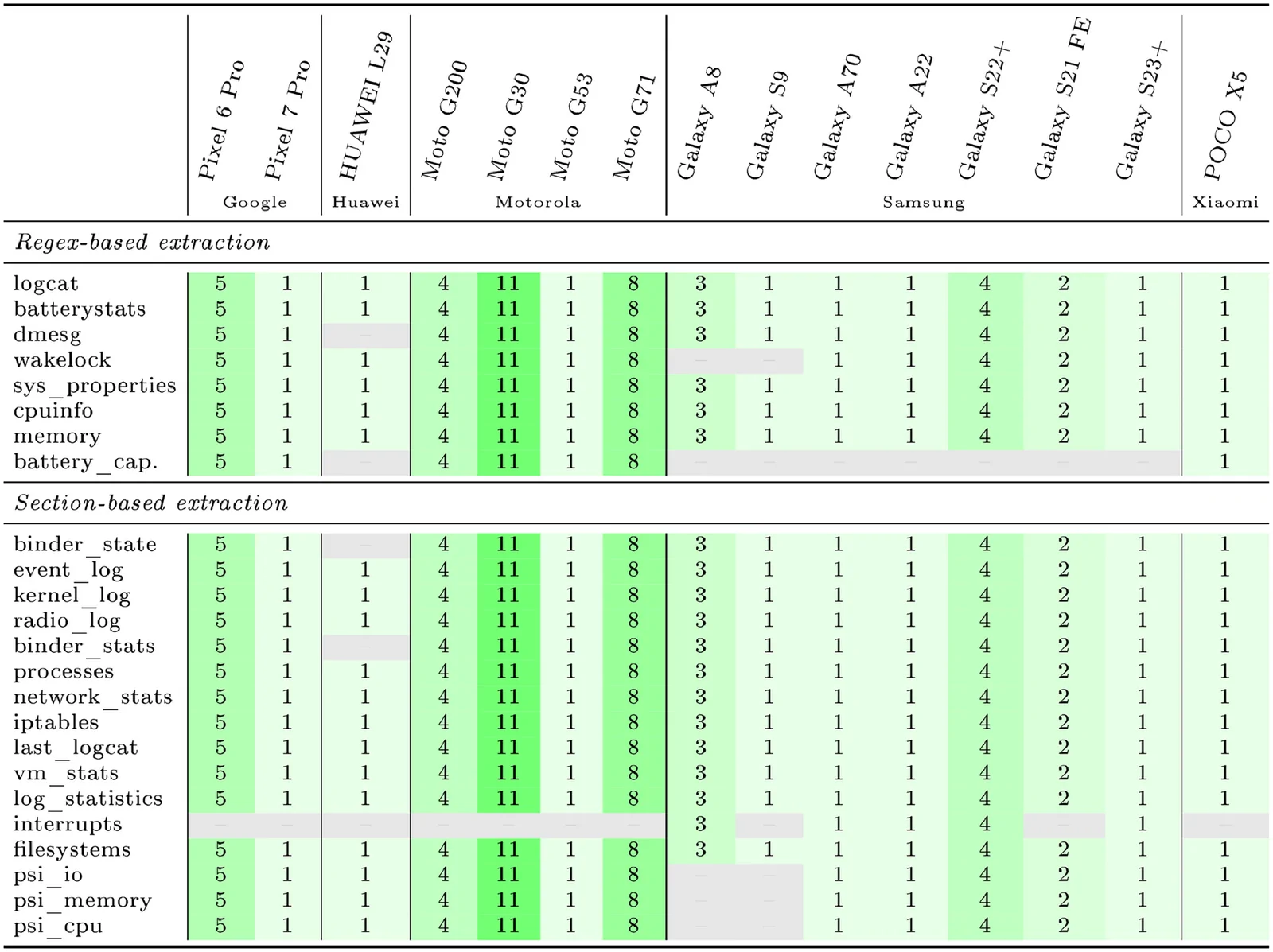
Block coverage matrix: number of extracted files per device and block type. Darker green indicates higher counts; dashes indicate absent blocks. Coverage of a block type is the number of captures in which the block is present divided by the 45 captures of the dataset.

### 3.4 Size comparison

The extraction pipeline reduces the 6.34 GiB of raw bugreport data to 1.14 GiB of structured block files, representing an 82.0% reduction in storage while preserving all analytically relevant content. The Drain parsing step generates an additional 1.74 GiB of structured CSV files. Fig. 5 illustrates these sizes.

**Fig. 5:**
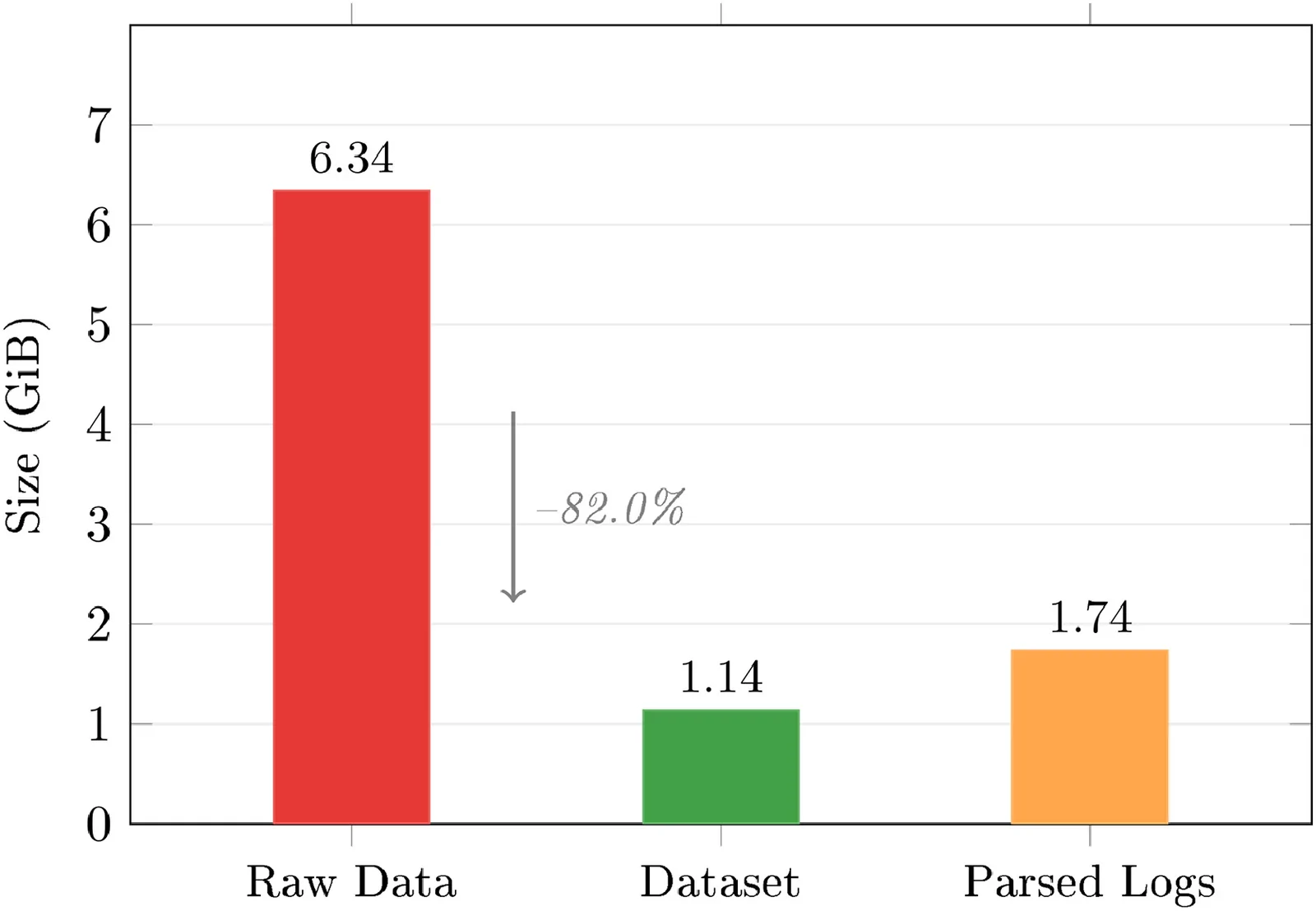
Size comparison between raw bugreport data, extracted dataset, and Drain-parsed log files.

### 3.5 Comparison with existing datasets

Table 3 compares ABRLog with related log datasets commonly used in the literature. While most existing datasets originate from server-side or supercomputer environments, ABRLog and ChiDroid [1] are among the few targeting Android mobile devices.

**Table 3:** Comparison of ABRLog with existing log datasets, grouped by domain. Sizes for ABRLog are binary (GiB); those of the other datasets are quoted as reported by their authors.

Domain	Dataset	Source	Systems	Log Types	Lines	Size
Mobile	**ABRLog (this work)**	Android bugreports	**15 devices**	24	∼10M	**1.14 GiB**
	ChiDroid [1]	Android logcat	2 devices	1	N/A	N/A
	Android Forensics [4]	Android diagnosis apps	1 vehicle	1	N/A	N/A
Server	Loghub v2.0 [2]	Various servers	14 systems	14	77M+	13 GB
	HDFS [10]	Hadoop cluster	1 cluster	1	11.2M	1.5 GB
HPC	BGL [11]	Supercomputer	1 system	1	4.7M	708 MB
	Thunderbird [11]	Supercomputer	1 system	1	211M	29 GB

While ABRLog is smaller in total volume than Loghub, it is unique in several respects: (1) it targets mobile devices rather than server infrastructure; (2) it provides 24 distinct block types from the same source captures rather than separate systems; (3) it covers five manufacturers and six Android versions, enabling cross-device and cross-manufacturer analysis; and (4) it preserves the per-device, per-capture organization that enables temporal analysis. Compared to other Android-focused datasets such as ChiDroid [1], ABRLog provides significantly broader device coverage (15 vs. 2 devices), multiple log types (24 vs. 1), and includes parsed log references.

### 3.6 Parsed log references

The Drain log parser [12] was applied to seven major block types, generating structured CSV files containing log templates and parsed log entries. A total of 310 block files (8596,594 lines) were parsed, yielding 212,071 log templates. Table 4 summarizes the parsing results per block type, and Fig. 6 illustrates the Drain input/output process. The parser consumes only non-empty lines, so the line counts in Table 4 may be marginally lower than the extracted line counts in Table 2; the only block affected is kernel_log, whose 382,252 extracted lines include 89 blank lines and therefore yield 382,163 parsed lines.

**Table 4:** Drain log parsing results per block type. Templates represent unique log event patterns discovered by the Drain algorithm. Line counts refer to non-empty lines submitted to the parser, which is why kernel_log reports 89 fewer lines than in Table 2.

Block Type	Files	Lines Parsed	Templates	Avg. Templates/File
logcat	45	4593,130	107,913	2398
batterystats	45	1472,653	37,561	835
dmesg	44	1137,480	40,748	926
event_log	45	407,697	4375	97
kernel_log	45	382,163	15,249	339
radio_log	45	380,865	5857	130
wakelock	41	222,606	368	9
**Total**	**310**	**8596,594**	**212,071**

**Fig. 6:**
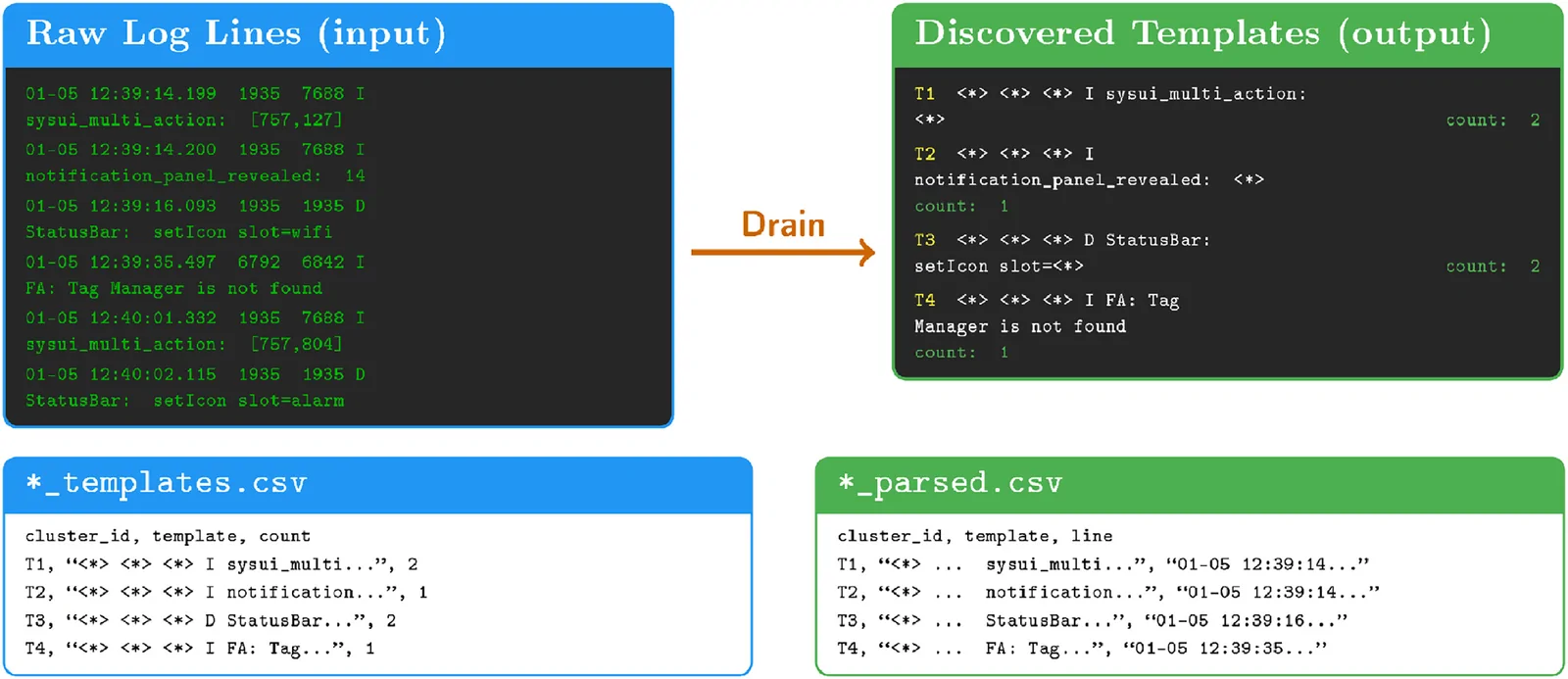
Illustration of the Drain log parsing process. Raw log lines (left) are processed by Drain to discover templates (right), where variable parts are replaced by 〈*〉 placeholders. Two CSV files are produced: a templates file with occurrence counts, and a parsed file that maps each line to its template.

The logcat block yields the highest number of templates (107,913), reflecting the diversity of system and application log messages. In contrast, wakelock produces only 368 templates across 41 files, indicating a highly regular event structure. The dmesg block shows the second-highest template density per file (926), consistent with the heterogeneous nature of kernel-level messages across different hardware configurations.

### 3.7 Data records

The complete dataset is publicly available on Zenodo (DOI: 10.5281/zenodo.21,892,365) as five ZIP archives and a README file. Table 5 describes each file. The archive ABRLog_SummaryData.zip holds the aggregated values behind every table and figure of this article as small CSV files, so that the reported numbers can be verified and reused without downloading the multi-gigabyte archives.

**Table 5:** Files available on the Zenodo repository.

File	Contents	Size (approx.)
ABRLog_SummaryData.zip	Aggregated values behind every table and figure of this article (CSV)	<1 MiB
ABRLog_RawData.zip	Raw bugreport files from 15 devices (45 captures)	6.3 GiB
ABRLog_Dataset.zip	Extracted log blocks (24 types, 1012 files)	1.2 GiB
ABRLog_ParsedLogs.zip	Drain-parsed CSV files (620 files: templates + parsed logs)	1.7 GiB
ABRLog_Scripts.zip	Python extraction, analysis, and parsing scripts	30 KB
README.md	Dataset documentation and usage instructions	—

The internal directory structure follows a consistent three-level hierarchy organized by device model and bugreport capture (Fig. 7).

**Fig. 7:**
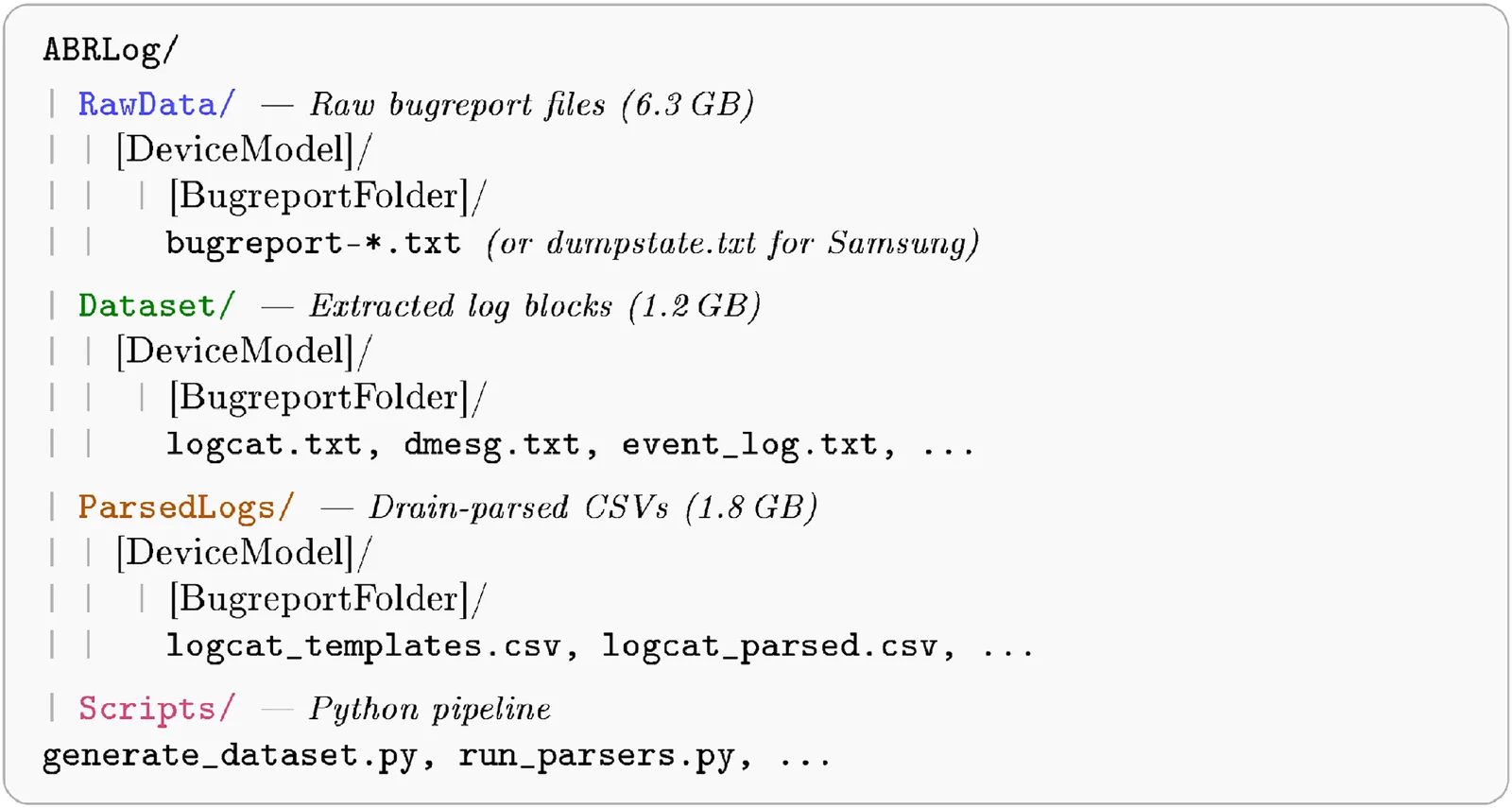
Directory structure of the ABRLog dataset on Zenodo.

File naming follows a deterministic convention across the three trees. The first level is the device folder, whose name is the device label used in Table 1 (for instance Samsung S22 or Pixel 6 Pro); Table 6 maps every device folder to its commercial model number and firmware build. The second level is the capture folder, which preserves the folder name produced by Android, of the form bugreport-[codename]-[build]-[YYYY-MM-DD-HH-MM-SS], so that the capture instant and the firmware are recoverable from the path itself. The third level holds the files: a raw capture in RawData/, one [block].txt file per extracted block type in Dataset/, and the pair [block]_parsed.csv and [block]_templates.csv in ParsedLogs/. Because the three trees share the same first two levels, a block file and its parsed counterparts are addressed by substituting only the root directory.

**Table 6:** Firmware build identifiers of the devices used in ABRLog, read from the ro.build.* properties of each capture.

Device folder	Model	Android	SDK	Build ID	Security patch	Captures
HUAWEI L29	DEL-LX9	12	31	HUAWEIDEL-L29	2021–10–05	1
Moto G200	moto g200 5G	12	31	S1RXS32.50–13–22	2023–09–01	1
Moto G200	moto g200 5G	12	31	S1RXS32.50–13–23	2023–11–01	3
Moto G30	moto g(30)	12	31	S0RCS32.41–10–19–14	2023–03–01	11
Motorola G53	moto g53 5G	14	34	U1TPS34.29–83–7–3–10	2025–12–01	1
Motorola G71	moto g71 5G	12	31	S2RUBS32.51–15–3–17	2023–11–01	8
Pixel 6 Pro	Pixel 6 Pro	13	33	TQ3A.230,605.010	2023–06–05	5
Pixel 7 Pro	Pixel 7 Pro	13	33	TQ3A.230,605.012	2023–06–05	1
Samsung A22	SM-A225M	13	33	TP1A.220,624.014	2023–08–01	1
Samsung A70	SM-A705MN	11	30	RP1A.200,720.012	2023–06–01	1
Samsung A8	SM-A730F	9	28	PPR1.180,610.011	2021–12–01	3
Samsung FE 21	SM-G990E	14	34	UP1A.231,005.007	2024–05–01	2
Samsung S22	SM-S906E	13	33	TP1A.220,624.014	2023–02–01	4
Samsung S23	SM-S916B	14	34	UP1A.231,005.007	2025–03–01	1
Samsung S9	SM-G9600	9	28	PPR1.180,610.011	2019–09–01	1
Xiaomi POCO X5	22111317PG	14	34	UKQ1.231,003.002	2025–02–01	1

**RawData** contains the original bugreport text files as collected from each device. **Dataset** contains one plain-text file per extracted block type per bugreport capture.

**ParsedLogs** contains two CSV files per parsed block:

• *_templates.csv with columns: cluster_id (integer), template (string with 〈*〉 placeholders for variable parts), and count (number of lines matching the template).
• *_parsed.csv with columns: cluster_id (integer matching the templates file), template (the matched template string), and line (the original raw log line).

### 3.8 Usage examples

The following examples illustrate how researchers can use ABRLog:

1. **Cross-manufacturer kernel analysis:** Load all dmesg.txt files, group by manufacturer, and compare template distributions to identify manufacturer-specific kernel behaviors.
2. **Log parser benchmarking:** Use the Drain-generated *_templates.csv as reference output and evaluate alternative parsers (e.g., Logram, BRAIN) on the corresponding Dataset/*.txt files.
3. **Anomaly detection:** Train models on logcat templates from multiple captures of the same device, then detect anomalous template sequences in held-out captures.

## 4 Experimental Design, Materials and Methods

### 4.1 Data collection

Bugreport captures were collected manually from each Android device using the built-in BugReport tool, accessible through the device’s developer options menu. Each capture produces a compressed archive containing the main bugreport text file along with supporting files (dumpstate logs, HAL debug information, protobuf data). The main bugreport file is a single large text file (typically 50–500 MB) containing all diagnostic information in a structured but heterogeneous format.

For Samsung devices, the main file is named dumpstate.txt; for other manufacturers, it follows the pattern bugreport-[device]-[build]-[timestamp].txt. The extraction scripts handle both naming conventions automatically.

No host-side adb tooling took part in the capture: the report is produced by the device itself and the resulting archive was transferred to the workstation over USB, so the output format depends only on the Android build of each device. Because bugreport section layout varies across API levels, Table 6 reports the exact firmware of every device, read from the ro.build.* properties of each capture (build identifier, security patch level and Android release), which allows any observed formatting difference to be traced back to a specific build. The captures span API levels 28 to 34.

Before publication, every capture was screened for personally identifiable information with a scripted procedure distributed with the dataset (scan_pii.py). The screening applies ten rules, of two kinds: four whose syntax is self-evident (e-mail addresses, Brazilian phone numbers, MAC addresses and Android Account records) and six anchored on the name of the field (IMEI/IMSI/MEID, ICCID/MSISDN, device serial numbers, Wi-Fi SSIDs, GPS coordinates and notification text), so that bare digit sequences such as timestamps and process identifiers are not misread as identifiers. Plain-text captures, the protobuf artifacts (incident reports and tombstones), and the two bugreport archives shipped as ZIP files were all covered.

Everything the screening reported as identifying a person, directly or indirectly, was then pseudonymized with sanitize_dataset.py, which imports its detection rules from the screening script so that the two cannot diverge. In total, 287,264 occurrences of 15,525 distinct identifiers were replaced: 9528 e-mail accounts (including WhatsApp identifiers, whose local part is a telephone number), 4336 MAC addresses, 1194 Wi-Fi network names, 307 notification texts, 74 account records, 62 telephone numbers, 11 IMEI/IMSI values, 8 GPS coordinates and 5 ICCID/MSISDN values.

Replacement is deterministic: each identifier is mapped through HMAC-SHA256 under a random key onto a pseudonym that preserves the shape of the original — digit counts, quoting, and the user@domain form. A given account is therefore represented by the same pseudonym in every file and on every device, so cross-capture correlation is retained, while the identity behind it cannot be recovered from the published data. Distinct originals yield distinct pseudonyms, which matters because log parsing measures precisely the variability between lines: collapsing different values onto a single placeholder would alter the statistic the dataset exists to support. Length-sensitive containers (protobuf) receive replacements of identical byte length, and their wire format was verified to remain parseable afterwards. Device serial numbers were deliberately preserved, as they identify the handset rather than a person. Re-running the screening over the published data reports pseudonyms only, and the table mapping originals to pseudonyms was not published.

All processing times reported in this article were measured on a workstation with a 12th-generation Intel Core i7–12700H processor (14 cores, 20 threads), 32 GB of RAM and a 1 TB NVMe solid-state drive, running Windows 11 (build 26,200) and Python 3.11. Both the extraction and the parsing stages run single-threaded, and the single-pass design of the extraction step makes its runtime scale linearly with the total size of the raw captures.

### 4.2 Block segmentation

Two complementary extraction approaches were implemented to maximize coverage of the diverse data present in bugreport files.

**Method 1: Line-by-line regular expression matching.** This method applies compiled regular expressions to each line of the bugreport file, collecting lines that match specific log formats. This approach is effective for log types with a consistent per-line format that may appear in multiple locations throughout the file. Eight block types are extracted using this method:

• dmesg: Kernel log messages matching [timestamp] message with optional priority prefix.
• logcat: System log entries in standard format MM-DD HH:MM:SS.mmm PID TID LEVEL TAG: message.
• batterystats: Battery statistics entries with duration-based timestamps.
• battery_capacity: Battery capacity and computed drain summaries.
• cpuinfo: Per-process CPU usage entries.
• memory: Memory information entries from /proc/meminfo.
• system_properties: Android system properties in [key]: [value] format.
• wakelock: Wake lock acquisition and release events.

The regular expressions were designed based on Android source code documentation available at Android Code Search (https://cs.android.com).

**Method 2: Section-based delimiter parsing.** Android bugreport files organize data into clearly demarcated sections identified by start and end markers (Fig. 8). A state-machine parser reads the file once and extracts all content between matching start and end delimiters. Sixteen block types are extracted using this method, including event_log, radio_log, kernel_log, processes, binder_state, binder_stats, network_stats, vm_stats, iptables, filesystems, log_statistics, and the PSI blocks.

**Fig. 8:**
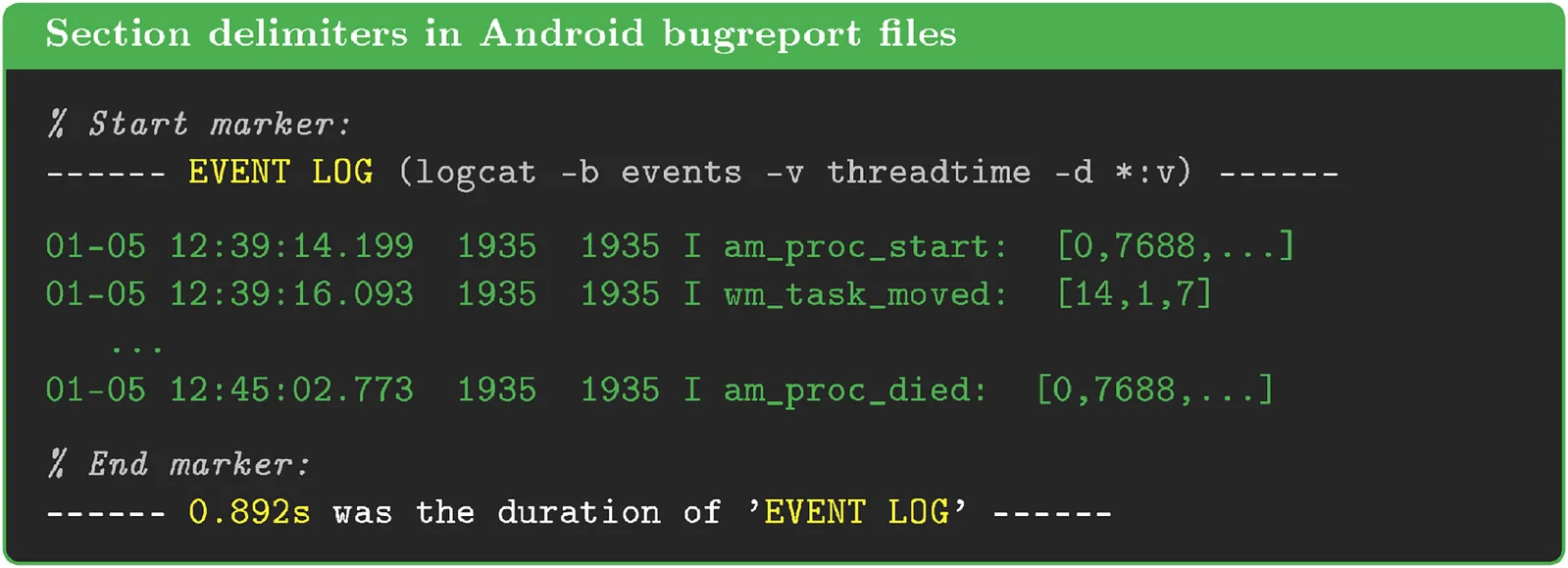
Example of section-based delimiter markers in a bugreport file. The start marker contains the section name and command; the end marker reports the extraction duration. All lines between the markers are captured as the block content.

Table 7 lists the complete set of extraction rules, enabling the segmentation stage to be reproduced without consulting the source code. Section rules capture the first occurrence of each section only, which avoids duplicating content when a bugreport repeats a header.

**Table 7:** Complete set of extraction rules used to build ABRLog: 8 regular expressions applied line by line and 17 section delimiters. Section rules match the bugreport header line —— KEYWORD … —— and capture every line until the next header. Long patterns are wrapped for display; the exact strings are in extraction_patterns.csv, distributed with the dataset.

Block type	Method	Rule
Dmesg	regex	^(<\d+>)?\[\s*\d+\.\d{6}\]
Cpuinfo	regex	^ *\+?(\d+\.?\d*)\% (\d+)/([^]+):
Batterystats	regex	^\s*\+(?:(\d+)d)?(?:(\d+)h)?(?:(\d+)m)?(?:(\d+ )s)?(?:(\d+)ms)? \(\d+\) (\d+) \w+ .*
battery_capacity	regex	^\s*Capacity: (\d+), Computed drain: \d+.*
Logcat	regex	^(\d{2}-\d{2} \d{2}:\d{2}:\d{2}.\d{3})(?:\s+[0–9A-Za-z]+)?\s+(\d+)\s+(\d+)\s+([A-Z])\s+(.+?) \s*: (.*)$
system_properties	regex	^\[(.*)\]: \[(.*)\]$
Memory	regex	^([^:]+):\s+(\d+) kB
Wakelock	regex	(\d{2}-\d{2} \d{2}:\d{2}:\d{2}.\d{3} - \d+ - ( REL|ACQ))
event_log	section	—— EVENT LOG … ——
radio_log	section	—— RADIO LOG … ——
kernel_log	section	—— KERNEL LOG … ——
stats_log	section	—— STATS LOG … ——
last_logcat	section	—— LAST LOGCAT … ——
Processes	section	—— PROCESSES AND THREADS … ——
network_stats	section	—— NETSTAT … ——
binder_stats	section	—— BINDER STATS … ——
binder_state	section	—— BINDER STATE … ——
vm_stats	section	—— VIRTUAL MEMORY STATS … ——
psi_cpu	section	—— PSI cpu … ——
psi_memory	section	—— PSI memory … ——
psi_io	section	—— PSI io … ——
Iptables	section	—— IPTABLES … ——
Filesystems	section	—— FILESYSTEMS & FREE SPACE … ——
Interrupts	section	—— INTERRUPTS … ——
log_statistics	section	—— LOG STATISTICS … ——

### 4.3 Log parsing with drain

The Drain algorithm [12] was applied to seven major block types to generate structured parsed logs. Drain uses a fixed-depth tree structure to efficiently parse log messages into templates by identifying the constant and variable parts of each log line. The following Drain3 configuration parameters were used: similarity threshold sim_th = 0.4, tree depth = 4, and maximum children per node max_children = 100. For each block file, Drain produces:

1. A *templates* file listing all discovered log templates with their occurrence counts.
2. A *parsed log* file mapping each original log line to its corresponding template cluster.

These parsed references enable researchers to benchmark other log parsing algorithms (e.g., Logram [13], BRAIN [14], NuLog [15]) against the Drain-generated output on real Android log data.

### 4.4 Implementation

The extraction and parsing pipeline is implemented in Python 3.11 and consists of three main components:

1. utils/extract_blocks.py: Defines the PATTERNS dictionary (8 compiled regular expressions) and the SECTION_PATTERNS dictionary (16 section keywords). Provides single-pass extraction functions.
2. generate_dataset.py: Discovers bugreport files, applies both extraction methods, and writes results to Dataset/. Supports incremental processing and command-line options for selecting specific patterns or sections.
3. run_parsers.py: Applies Drain (via drain3 v0.9.11) to the extracted blocks and generates parsed log CSV files in ParsedLogs/.

Dependencies are listed in requirements.txt and include matplotlib, seaborn, pandas, tqdm, and drain3. The pipeline processes the full dataset (6.34 GiB raw data) in approximately 2.5 min for block extraction and 16 min for Drain parsing.

### 4.5 Extensibility

To add a new regex-based block type, add an entry to PATTERNS in extract_blocks.py. For section-based blocks, add an entry to SECTION_PATTERNS. Re-running the extraction script generates the new block files for all bugreports. The parsing step can similarly be extended to additional parsers.

## Limitations

• The dataset covers 15 devices from five manufacturers but does not represent all Android device categories (e.g., tablets, wearables) or all major manufacturers (e.g., OnePlus, Oppo, Vivo).
• Android bugreport formats are not standardized across manufacturers. Samsung devices include vendor-specific sections (e.g., INTERRUPTS, NFC SYSTEM LOG) absent from other manufacturers, resulting in uneven block coverage.
• The dmesg regex-based extraction and the kernel_log section-based extraction provide overlapping but not identical content, as the regex matches kernel-format lines throughout the entire file while the section extraction captures only the demarcated kernel log section.
• Bugreport captures were collected during normal device usage without controlled fault injection. Researchers using this dataset for anomaly detection should be aware that the logs primarily represent normal operating conditions.
• The Drain-generated parsed logs serve as reference output, not as manually verified ground truth. Template accuracy may vary across block types and device manufacturers.
• Account addresses, telephone numbers, network identifiers (MAC and SSID), subscriber identifiers, GPS coordinates and notification text were pseudonymized before publication. Replacements are deterministic and shape-preserving, so log structure and cross-capture correlation are retained, but analyses that depend on the literal values of these fields — recovering actual network names, for instance — cannot be carried out on ABRLog. Device serial numbers were preserved.

## Ethics Statement

The authors have read and follow the ethical requirements for publication in Data in Brief and confirm that the current work does not involve human subjects, animal experiments, or any data collected from social media platforms. Bugreport captures were collected from devices owned by the research team members. Personal data present in the raw logs — including account addresses, telephone numbers, network identifiers, and notification content, some of it relating to third parties who appear in the logs of the researchers' own devices — were located by a scripted screening procedure and pseudonymized before publication, as described in Experimental Design, Materials and Methods. The published dataset therefore does not expose personally identifiable information; the screening and pseudonymization scripts are distributed with the data, and the mapping between original values and pseudonyms was not published.

## CRediT Author Statement

**João Alfredo Bessa:** Conceptualization, Methodology, Data Curation, Writing. **Ricardo Miranda Filho:** Software, Validation, Data Curation, Writing. **Rosiane de Freitas:** Supervision, Writing – Review & Editing.
